# CRISPR/Cas9-based generation of a recombinant double-reporter pseudorabies virus and its characterization in vitro and in vivo

**DOI:** 10.1186/s13567-021-00964-4

**Published:** 2021-06-26

**Authors:** Peng-Fei Fu, Xuan Cheng, Bing-Qian Su, Li-Fang Duan, Cong-Rong Wang, Xin-Rui Niu, Jiang Wang, Guo-Yu Yang, Bei-Bei Chu

**Affiliations:** College of Veterinary Medicine, Henan Agricultural University, Zhengzhou, 450046 Henan China

**Keywords:** Pseudorabies virus, Firefly luciferase, EGFP, CRISPR/Cas9, Imaging in vivo, 25-hydroxycholesterol

## Abstract

Pseudorabies, caused by pseudorabies virus (PRV) variants, has broken out among commercial PRV vaccine-immunized swine herds and resulted in major economic losses to the pig industry in China since late 2011. However, the mechanism of virulence enhancement of variant PRV is currently unclear. Here, a recombinant PRV (rPRV HN1201-EGFP-Luc) with stable expression of enhanced green fluorescent protein (EGFP) and firefly luciferase as a double reporter virus was constructed on the basis of the PRV variant HN1201 through CRISPR/Cas9 gene-editing technology coupled with two sgRNAs. The biological characteristics of the recombinant virus and its lethality to mice were similar to those of the parental strain and displayed a stable viral titre and luciferase activity through 20 passages. Moreover, bioluminescence signals were detected in mice at 12 h after rPRV HN1201-EGFP-Luc infection. Using the double reporter PRV, we also found that 25-hydroxycholesterol had a significant inhibitory effect on PRV both in vivo and in vitro. These results suggested that the double reporter PRV based on PRV variant HN1201 should be an excellent tool for basic virology studies and evaluating antiviral agents.

## Introduction

Pseudorabies virus (PRV), belonging to the *Herpesviridae* family, subfamily *Alphaherpesvirinae*, genus *Varicellovirus*, is the aetiological agent of pseudorabies and was first described in 1813 [1–3]. PRV is a neurotropic herpesvirus that can establish latent infection in nerve cells [4–6]. This virus can cause severe encephalitis in juvenile pigs [7] and various non-native hosts [8–10]. In non-native hosts such as mice, PRV infection is always lethal [11]. Owing to the use of the PRV vaccine strain Bartha-K61 from Hungary, which contains a complete deletion of the regions encoding glycoprotein E (gE) and US9 and partial deletion within the gI and US2 coding regions, pseudorabies (PR) was well controlled on most pig farms in China between the 1990s and 2011 [12]. However, in late 2011, PR re-emerged in many pig herds immunized with the Bartha-K61 PRV vaccine in North China and spread rapidly across various provinces [13, 14]. Novel PRV variants have been found in the swine population of China [13, 15–20]. In addition, some human cases of viral endophthalmitis and encephalitis caused by PRV infection have been reported in China [21–25]. Given the urgency of the epidemic and PRV’s potential threat to human health, further research on PRV variants is critical.

Viral reverse genetics technology plays an important role in PRV vaccine development and pathogenicity research [26–28]. The genome of PRV is a linear double-stranded DNA approximately 150 kilobases kb in length, encoding ~70 proteins [1]; it includes many nonessential regions for replication in cell culture, such as the TK, PK, gI, gE, gC and gG genes [2, 29, 30]. These characteristics allow PRV to accommodate several kb of foreign DNA without affecting its stability. The traditional methods for editing PRV, homologous recombination (HR) and bacterial artificial chromosome (BAC) construction, are both laborious and time-consuming [31–33]. Clustered regulatory interspaced short palindromic repeats/CRISPR-associated protein 9 (CRISPR/Cas9) is an adaptive immune mechanism that evolved in archaea and bacteria [34]. This system has been developed as a powerful DNA engineering tool in diverse organisms. In this study, a recombinant virus (rPRV HN1201-EGFP-Luc) for stable expression of enhanced green fluorescent protein (EGFP) and firefly luciferase was constructed on the basis of the PRV variant HN1201 by using a two sgRNA CRISPR/Cas9 system and was applied to investigate the inhibitory effect of 25-hydroxycholesterol (25-HC) on PRV proliferation in PK-15 cells and in mice. The double reporter PRV carrying EGFP and firefly luciferase in this study enabled convenient quantification and tracing of PRV in vitro and in vivo.

## Materials and methods

### Mice

The Animal Care Committee of Henan Agricultural University (Zhengzhou, People’s Republic of China) approved this study (Approval Number 11-0085), and all animals were maintained in a specific-pathogen-free animal facility according to the related ethical regulations at Henan Agricultural University and the guide for the care and use of laboratory animals.

Female 6–8-week-old BALB/c mice were purchased from the Center of Experimental Animal of Zhengzhou University (Zhengzhou, China) and maintained in a specific pathogen-free animal facility according to the guide for the care and use of laboratory animals and the related ethical regulations at Henan Agricultural University.

### Cell lines and viruses

PK-15, ST and 3D4/21 cells were cultured in Dulbecco’s modified Eagle’s medium (DMEM) (Gibco, Grand Island, NY, USA) supplemented with 10% (v/v) foetal bovine serum (FBS; Gibco) at 37 °C with 5% CO_2_. The PRV variant HN1201 (GenBank Accession No. KP722022) was a kind gift from Ke-gong Tian at the College of Veterinary Medicine, Henan Agricultural University [16].

### Generation of the donor plasmid and double sgRNA CRISPR/Cas9 plasmid

To construct the plasmid pX459M-sgRNA1-sgRNA2, we obtained small guide RNA1 (sgRNA1) targeting PRV gI from the annealing product of sgRNA1-Fwd and sgRNA1-Rev oligos and inserted it into the pX459M vector (Addgene, Watertown, MA, USA) at the *Bbs*I site to produce the recombinant plasmid pX459M-sgRNA1. The sgRNA2-Fwd and sgRNA2-Rev sequences were annealed to form sgRNA2 targeting PRV gE and cloned into the pEZ-Guide-XH vector at the *Bbs*I site. Then, sgRNA2 was obtained from the pEZ-Guide-XH vector via the *Xho*I and *Hin*dIII sites and inserted into the plasmid pX459M-sgRNA1 to yield the recombinant plasmid pX459M-sgRNA1-sgRNA2. Two sgRNAs for editing PRV were used in this study and are listed in Table 1. There were no potential off-target regions for sgRNA1 and sgRNA2 in the PRV genome, as determined by using CRISPR [35].

**Table 1:** **Sequences of the primers and sgRNAs utilized in this study**

Primers and sgRNAs	Sequences (5'–3')
US 6/7-F	5'-GAATTCCGGCGTGAACATCCTCACCGACT-3'
US 6/7-R	5'-GATATCGCAGCGTCCCGTCTATCGTCAGGT-3'
US 8/9-F	5'-GGATCCGCCCACGCACGAGGACTACTACGA-3'
US 8/9-R	5'-GCTAGCGGTGGAGGCGGTGGAGAAGAAGAG-3'
sgRNA1-F	5'-CACCGTCGTGCCACGATCCGACGA-3'
sgRNA1-R	5'-AAACTCGTCGGATCGTGGCACGAC-3'
sgRNA2-F	5'-CACCGTACAGCCCCGACTCGTCCG-3'
sgRNA2-R	5'-AAACCGGACGAGTCGGGGCTGTAC-3'
U6-F	5'-CTATTTCCCATGATTCCTTCA-3'
CAG-screen R	5'-GTACTGGGCACAATGCCAG-3'
Porcine actin-F	5'-CTGAACCCCAAAGCCAACCGT-3'
Porcine actin-R	5'-TTCTCCTTGATGTCCCGCACG-3'
Porcine IFN-β-F	5'-AGTTGCCTGGGACTCCTCAA -3'
Porcine IFN-β-R	5'-CCTCAGGGACCTCAAAGTTCAT-3'
Porcine IL-1β-F	5'-CCATCCACTGAGCCAGCCTT-3'
Porcine IL-1β-R	5'-TGCCAAGGACAGAGGACTGC-3'
Luc-F	5'-ATGGAAGACGCCAAAAACATAAAG-3'
Luc-R	5'-TTACACGGCGATCTTTCCGCCCT-3'

To obtain the donor plasmid pUC57-US6/7-EGFP-Luc-US8/9, we synthesized DNA fragments containing *Eco*RI, *Eco*RV, *Pac*I, *Xho*I, *Bam*HI, *Nhe*I and *Hin*dIII sites and inserted them into the pUC57 vector between the *Eco*RI and *Hin*dIII sites to replace the multiple cloning site of the pUC57 vector. The right HR arm (including the partial US8 gene and the entire US9 gene of PRV HN1201) was amplified from the PRV HN1201 genome with the US 8/9-F/R primer set and inserted into the pUC57 vector between the *Bam*HI and *Nhe*I sites to construct the recombinant plasmid pUC57-US8/9. The firefly luciferase expression cassette was removed from the pGL3-Control vector with *Xho*I and *Bam*HI and inserted into the plasmid pUC57-US8/9, thus generating the recombinant plasmid pUC57-Luc-US8/9. Then, the EGFP expression cassette was released from the CBh-EGFP vector via the *Pac*I site and cloned into the recombinant plasmid pUC57-Luc-US8/9 to generate the recombinant plasmid pUC57-EGFP-Luc-US8/9. The left HR arm (including the partial US6 gene and partial US7 gene of PRV HN1201) was amplified from the PRV HN1201 genome with the US 6/7-F/R primer set and then inserted into the recombinant plasmid pUC57-EGFP-Luc-US8/9 between the *Eco*RI and *Eco*RV sites to obtain the donor plasmid pUC57-US6/7-EGFP-Luc-US8/9. Sequences of all plasmids constructed in the study were confirmed by Sanger sequencing.

### Generation of double reporter PRV

The PRV HN1201 genome was extracted as previously described [15, 36]. A total of 2 μg of donor plasmid pUC57-US6/7-EGFP-Luc-US8/9 was cotransfected into PK-15 cells together with 1 μg of two-sgRNA CRISPR/Cas9 plasmid pX459M-sgRNA1-sgRNA2 and 1 μg of the PRV HN1201 genome by using Lipofectamine 2000 (Invitrogen, USA) according to the manufacturer’s instructions. EGFP was detected by using a standard FITC filter-equipped fluorescence microscope (Nikon Eclipse TS100) at 2–3 days after cotransfection. When cytopathic effects (CPEs) appeared, the cells were collected. The cell lysate was serially diluted 10^–1^–10^–8^ fold and used to infect PK-15 cell monolayers in a 96-well cell culture plate. The recombinant virus was purified from the 96-well cell culture plate with an endpoint dilution assay under an inverted fluorescence microscope. After three virus purification steps and further luciferase assays, the recombinant virus was obtained and named rPRV HN1201-EGFP-Luc. The genomic DNA of rPRV HN1201-EGFP-Luc was extracted from cell lysates to amplify the expected fragment (including the left HR arm, the EGFP expression cassette, the firefly luciferase expression cassette and the right HR arm) with the primers US 6/7-F and US 8/9-R (Table 1), and PCR products were sequenced for identification of the recombinant virus.

### PCR and qPCR

PK-15 cells were harvested at specific times after PRV HN1201 or rPRV HN1201-EGFP-luc infection, and then, total RNA was extracted from PK-15 cells by using TRIzol reagent (9108, TaKaRa). cDNA was synthesized from the total RNA with a PrimeScript RT reagent Kit (RR047A, TaKaRa). qPCR was performed in triplicate by using SYBR Premix Ex Taq (RR820A, TaKaRa) according to the manufacturer’s instructions, and data were normalized to the level of β-actin expression in each sample. Melting curve analysis indicated the formation of a single product in all cases. Porcine IL-β, IFN-β and actin gene expression was detected with the primers porcine IL-β-F/R, IFN-β-F/R and actin-F/R (Table 1), and the 2^−ΔΔCt^ method was used to calculate relative expression changes. PCR was performed with PrimeSTAR^®^ HS DNA Polymerase (R010A, TaKaRa) and the primers Luc-F/R, PRV US6/7-F/R and PRV US8/9-F/R (Table 1) for the Luc gene and PRV US6/7 and PRV US8/9 genes.

### Western blotting

To examine the expression of viral proteins and firefly luciferase protein, we infected PK-15 and ST cells with rPRV HN1201-EGFP-luc or PRV HN1201 at a multiplicity of infection (MOI) of 0.1 for 24 h. Cells were collected in lysis buffer (50 mM Tris–HCl, pH 8.0, 150 mM NaCl, 1% Triton X-100, 1% sodium deoxycholate, 0.1% SDS and 2 mM MgCl_2_) supplemented with protease and phosphatase inhibitor cocktail (HY-K0010 and HY-K0022, MedChemExpress). The protein concentrations in the lysates were quantified with a BCA Protein Assay Kit (DingGuo, Beijing, China) and detected with a microplate reader (Awareness Technology, Inc., Palm City, FL, USA). Protein samples (30 μg) were separated by SDS-PAGE and transferred to nitrocellulose membranes (ISEQ00010, Millipore), which were incubated in 5% nonfat milk (A600669, Sangon) for 1 h at room temperature. The membrane was incubated with anti-firefly luciferase antibody (1:3000) (ab185924, Abcam), rabbit antiserum against PRV gE (1:500) (generated by immunizing a rabbit with purified recombinant gE), anti-PRV gB mouse monoclonal antibody (1:2000) (prepared and stored by our laboratory) or anti-β-actin antibody (1:5000) (A1978, Sigma-Aldrich) at 4 °C overnight and then incubated with horseradish peroxidase (HRP)-conjugated secondary antibody (715-035-150 or 711-035-152, Jackson ImmunoResearch Laboratories) for 1 h. Immunoblotting results were visualized with Luminata Crescendo Western HRP Substrate (WBLUR0500, Millipore) on a GE AI600 imaging system.

### Firefly luciferase assay

The luciferase assay was performed with a Dual-Luciferase^®^ Reporter Assay System (E1960, Promega) according to the manufacturer’s instructions. Briefly, growth medium was removed from the cultured cells in a 12-well culture plate, and a sufficient volume of 1 × phosphate-buffered saline (PBS) was gently applied to rinse the bottom of the culture vessel. After 250 μL of 1 × Passive Lysis Buffer (PLB) was added to the wells, the plate was shaken gently at room temperature for 15 min, and the cell lysate was transferred to a white 96-well test plate containing 100 μL of Luciferase Assay Reagent II (LAR II). The relative light units (RLU) were detected with a Thermo Scientific Fluoroskan Ascent FL (Thermo Fisher, USA).

### Flow cytometry assay

For the EGFP reporter assay, PK-15 cells were pretreated with 25-HC at the indicated concentration for 4 h and then infected with rPRV HN1201-EGFP-Luc (MOI = 0.01) and simultaneously treated with 25-HC at the indicated concentration for 18 h. Cells were digested with trypsin–EDTA (25200072, Gibco), collected by centrifugation and suspended in 1 × PBS. The percentage of EGFP-positive cells was measured by flow cytometry on a CytoFLEX instrument.

### Assessment of genetic stability

The rPRV HN1201-EGFP-Luc vector was propagated in PK-15 cells. PK-15 cells at 90% confluence grown in 10 cm dishes were infected with rPRV HN1201-EGFP-Luc (MOI = 0.01) per cell. After incubation at 37 °C for 1 h, the cells were washed, and fresh 2% (v/v) foetal bovine serum medium was added. When CPEs appeared in most cells, the viruses were harvested and titrated to determine the TCID_50_ values. To test the genetic stability of rPRV HN1201-EGFP-Luc, we serially passaged the virus in PK-15 cells 20 times, denoted P1 to P20.

### Plaque assays

Plaque assays were performed by infecting confluent PK-15 cells in six-well plates with the indicated viruses. The cells were washed three times with 1 × PBS and infected with an appropriate dilution of PRV HN1201 or rPRV HN1201-EGFP-Luc containing the same gene copy number for 1 h at 37 °C and 5% CO_2_. The viral inoculum was removed, and the cells were washed with 1 × PBS three times. Then, the cell monolayers were overlaid with DMEM (Gibco, Grand Island, NY, USA) containing 1% methylcellulose (M8070, Solarbio) and 2% FBS (10099-141C, Gibco) at 37 °C and 5% CO_2_ for 36 h. Next, the medium was removed, and the cell monolayer was fixed with 4% paraformaldehyde (P1110, Solarbio) for 1 h and stained with 1% crystal violet (C8470, Solarbio); plaques were then counted. The number of rPRV HN1201-EGFP-Luc plaques was normalized to the number of PRV HN1201 plaques to detect the effect of gI/gE gene knockout on PRV packaging. The plaque diameters were measured from scanned images by using ImageJ.

### Mouse survival study

Mice were used to evaluate the virulence of the recombinant virus rPRV HN1201-EGFP-Luc and the parental strain PRV HN1201. Forty 6-week-old healthy female BALB/c mice were randomly divided into five groups of eight. The mice (*n* = 8) in groups 1–4 were inoculated intramuscularly (I.M.) in the left leg with 10^2.5^ TCID_50_ and 10^6.5^ TCID_50_ rPRV HN1201-EGFP-Luc or 10^2.5^ TCID_50_ and 10^6.5^ TCID_50_ PRV HN1201. The mice in group 5 were inoculated with DMEM as an uninfected control. After inoculation, the survival of the mice was recorded every day for 8 days.

### Viral infection and in vivo imaging

For in vivo rPRV HN1201-EGFP-Luc infection, 6-week-old mice (five mice per group) were anaesthetized with isoflurane and inoculated I.M. with rPRV HN1201-EGFP-Luc (10^6.5^, 10^5.5^, 10^4.5^, 10^3.5^ or 10^2.5^ TCID_50_ per mouse). Mice (*n* = 5) inoculated with 2% FBS DMEM served as an uninfected control. The rPRV HN1201-EGFP-Luc distribution in mice was measured by whole body imaging with an IVIS Lumina III (Perkin Elmer) instrument after intraperitoneal injection of D-luciferin sodium salt (HY-12591, MCE) (3 mg per mouse) at the indicated times.

### The 25-HC antiviral tests in vivo and in vitro

PK-15 cells were seeded in 12-well plates (2 × 10^5^ cells/well) and incubated at 37 °C with 5% CO_2_ for 12 h. PK-15 cells were infected with rPRV HN1201-EGFP-Luc (MOI = 0.01) per cell and 25-HC at the corresponding concentration for 18 h after pretreatment with 25-HC (HY-113134, MCE) (0.1 μM, 0.3 μM, 1 μM, 3 μM or 10 μM) for 4 h. DMSO solvent-only control was used in the DMSO group (1 μL of DMSO was added to 1000 μL of cell culture medium). The inhibitory effects of 25-HC on PRV proliferation in PK-15 cells were evaluated with firefly luciferase assays, flow cytometry assays and fluorescence analysis.

Six-week-old mice were randomly divided into a 25-HC group and DMSO group (three mice per group). For the 25-HC group, 25-HC (10 mg/kg) was administered to mice by intraperitoneal injection for 2 consecutive days (once per day). For the DMSO group, mice received the same volume of DMSO. Twelve hours after the last injection of 25-HC, all mice were injected I.M. in the left leg with rPRV HN1201-EGFP-Luc (1 × 10^4.5^ TCID_50_ per mouse). The rPRV HN1201-EGFP-Luc distributions in mice were measured with whole animal bioluminescence imaging by using an IVIS Lumina III (Perkin Elmer) instrument after intraperitoneal injection of D-luciferin sodium salt (HY-12591, MCE) (3 mg per mouse) at 24 h after viral infection.

To determine the suitability of 25-HC as a curative agent, we administered 25-HC after infection. In the experimental animal groups, the injection dosage and methods for 25-HC and DMSO were the same as above, except that each mouse was first infected with 1 × 10^4.5^ TCID_50_ rPRV HN1201-EGFP-Luc and then injected with 25-HC or DMSO 6 h and 12 h after virus infection. The rPRV HN1201-EGFP-Luc distributions in mice were measured with whole animal bioluminescence imaging 24 h after viral infection by using an IVIS Lumina III (Perkin Elmer) instrument after intraperitoneal injection of D-luciferin sodium salt (HY-12591, MCE) (3 mg per mouse).

### Statistical analysis

Data were obtained from at least three independent experiments for the quantitative analyses and are expressed as the mean ± standard errors of the means. All statistical analyses were performed with a *t-*test or one-way analysis of variance. Significant differences were accepted at *P* values of  < 0.05, < 0.01 and < 0.001 versus the corresponding controls. For mouse survival studies, Kaplan–Meier survival curves were generated and analysed for statistical significance.

## Results

### Generation of rPRV HN1201-EGFP-Luc

**Figure 1:**
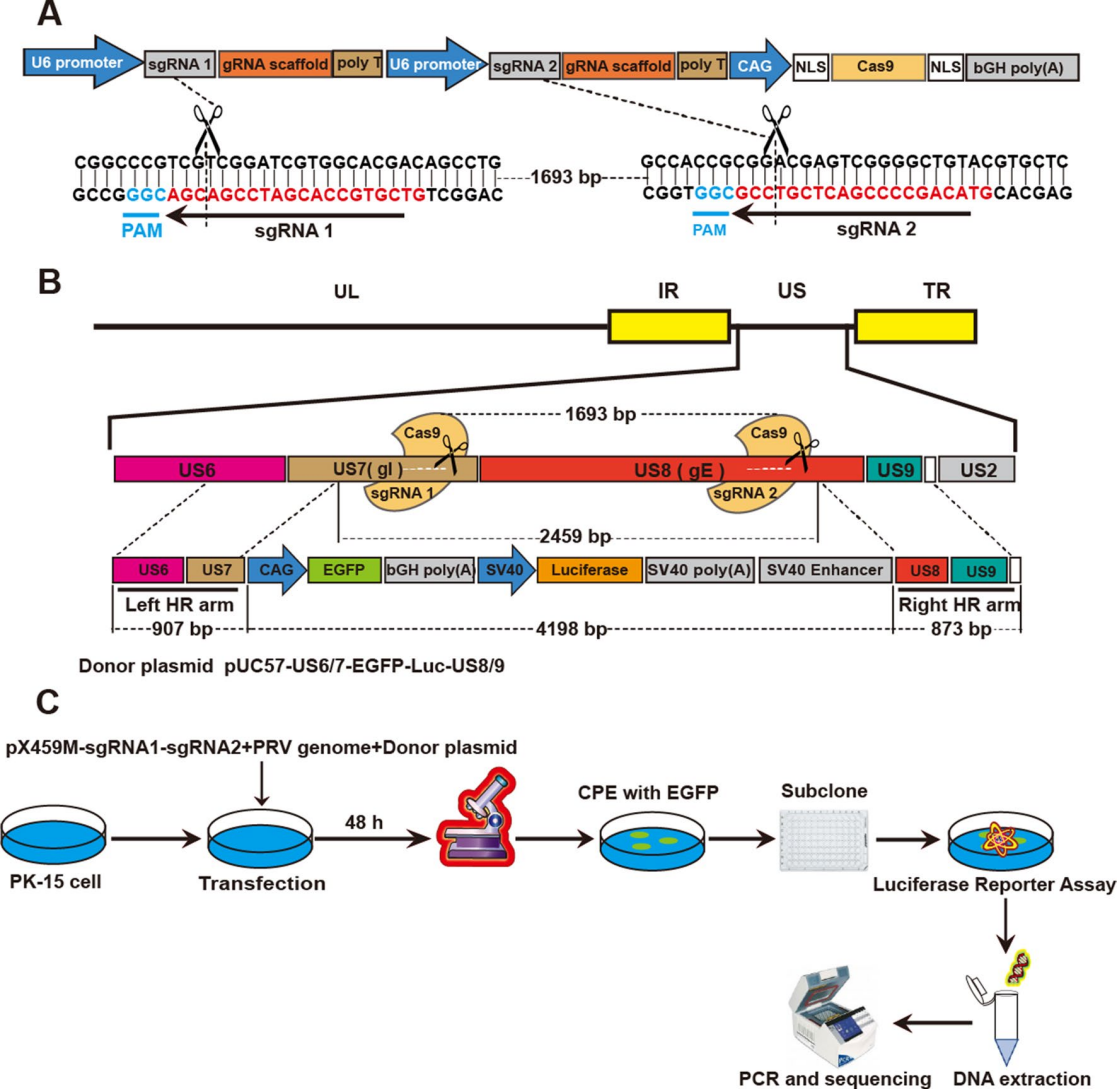
**The double-sgRNA CRISPR/Cas9 system and the protocol used to generate the recombinant virus rPRV HN1201-EGFP-Luc**. **A** Schematic diagrams of the double -sgRNA CRISPR/Cas9 system and the CRISPR/Cas9 cleavage positions. **B** Diagrams showing the PRV HN1201 genome and the donor plasmid. In the donor plasmid, the left homologous recombination arm (left HR arm) and the right HR arm are located upstream and downstream of the sgRNA1 and sgRNA2 target sites, respectively. There were 1693 base pairs (bp) between the two sgRNA target sites. After the PRV genome is cut at the two sgRNA target sites and homologous recombination occurs, the PRV genome loses a 2459 bp DNA fragment, and the cassette containing the EGFP and luciferase genes is knocked in at the DNA deletion sites. **C** Diagram depicting the protocol used to obtain and purify recombinant PRV for the expression of EGFP and luciferase genes. A mixture of PRV HN1201 genomes, Cas9/sgRNAs and donor plasmid was cotransfected into PK-15 cells. CPEs with EGFP were observed 2–4 days after transfection. Cells and media were collected after three freeze–thaw cycles and then inoculated into cells in 96-well plates after serial dilutions to obtain single viral clones. Subcloned viruses were subjected to luciferase assays and sequence analysis.

**Figure 2:**
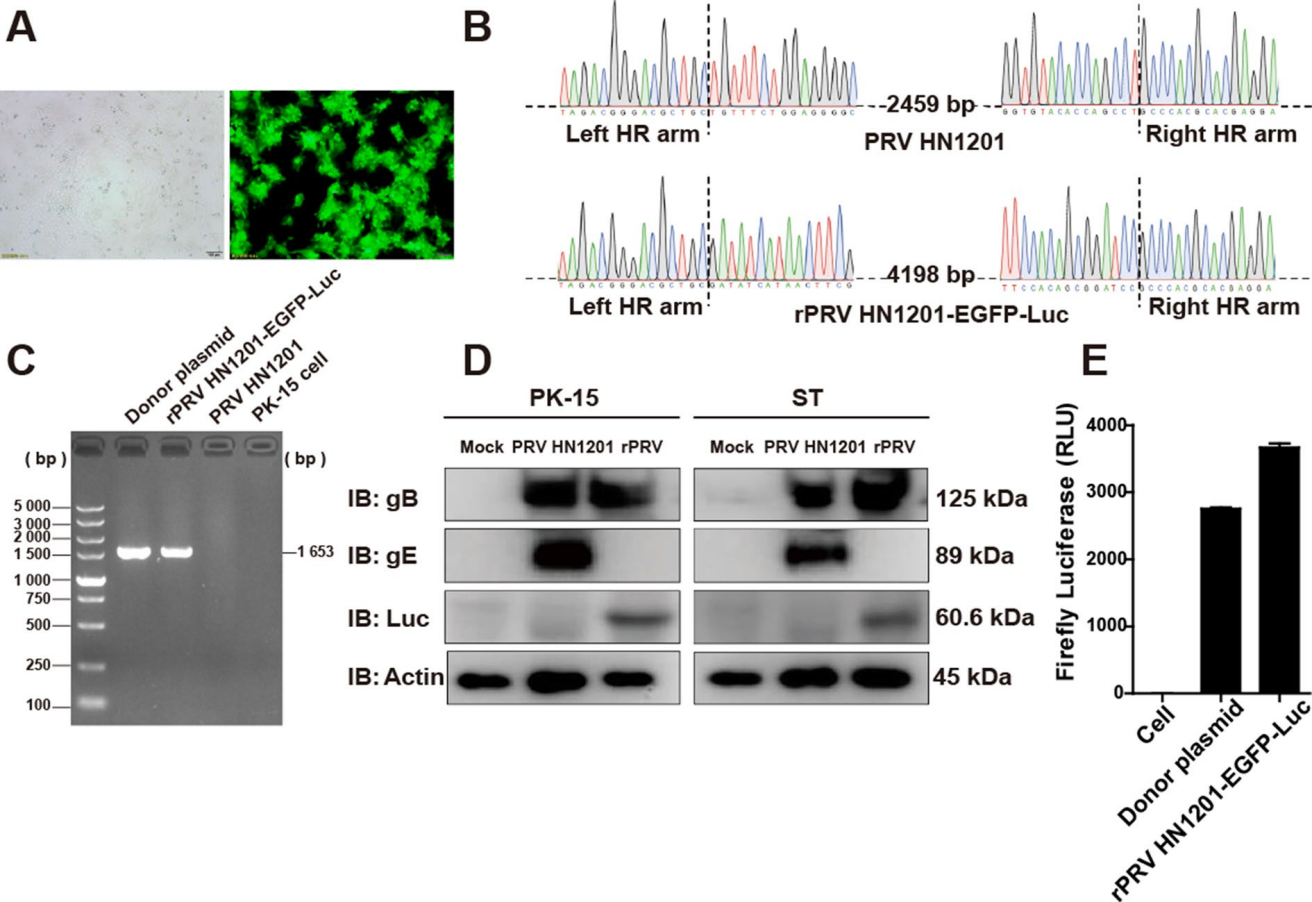
**Identification of the recombinant virus rPRV HN1201-EGFP-Luc.** **A** PK-15 cells were infected with purified recombinant rPRV HN1201-EGFP-Luc virus and visualized by fluorescence and bright field microscopy. **B** Sequence chromatogram showing the DNA sequences of PRV HN1201 (top) and rPRV HN1201-EGFP-Luc (bottom) at the genetic homologous recombination sites. **C** Cells were infected with rPRV HN1201-EGFP-Luc (MOI = 0.1) for 12 h. The mRNA of firefly luciferase in the cells was detected with RT-PCR. **D** PK-15 cells (left) and ST cells (right) were either mock infected or infected with PRV HN1201 or rPRV HN1201-EGFP-Luc (MOI = 0.1) for 24 h and then lysed for immunoblotting analysis with antibodies against firefly luciferase and the viral proteins gB and gE. β-Actin was used as the loading control. **E** PK-15 cells were infected with rPRV HN1201-EGFP-Luc (MOI = 0.1) for 24 h and then lysed for luciferase assays. PK-15 cell lysate was used as a negative control, and PK-15 cells transfected with donor plasmid lysate were used as a positive control. This experiment was performed three times, and the results are shown as the mean ± SD.

PRV glycoprotein I (gI) and glycoprotein E (gE), two adjacent proteins, are nonessential for PRV replication in vitro [10, 37]. Hence, this region was chosen for insertion of the EGFP and firefly luciferase reporter genes. First, a two sgRNA CRISPR/Cas9 plasmid system (Figure 1A) was constructed to improve the efficiency of PRV genome editing. Subsequently, a donor plasmid containing both EGFP and firefly luciferase expression cassettes flanked by homologous arms was constructed, in which the EGFP expression cassette was under the control of the CAG promoter (a synthetic promoter composed of a CMV enhancer and chicken β-actin promoter), and the firefly luciferase expression cassette was under the control of the SV40 promoter (Figure 1B). Next, the recombinant virus rPRV HN1201-EGFP-Luc (Figure 1C) was generated by cotransfection of the donor plasmid pUC57-US6/7-EGFP-Luc-US8/9, plasmid PX459M-sgRNA1-sgRNA2 and genomic DNA of the parental strain PRV HN1201 into PK-15 cells. Approximately 48 h post-infection (hpi), clear CPEs were observed, and recombinant viruses were collected and purified with endpoint dilution assays under a fluorescence microscope until all clones showed green fluorescence (Figure 2A). Further DNA sequencing analysis revealed that the viral genomes were cleaved at two sgRNA target sites and repaired by the homology-directed repair (HDR) pathway, thus resulting in a 2459 bp DNA fragment with the PRV gI and gE genes deleted and cassettes containing EGFP and firefly luciferase genes (4198 bp) inserted at the DNA deletion sites (Figure 2B).

### Cidentification of the reombinant virus rPRV HN1201-EGFP-Luc

To confirm the expression of firefly luciferase in PK-15 cells, we extracted total RNA from the cells infected with rPRV HN1201-EGFP-Luc after 12 h and performed RT-PCR analysis for the presence of firefly luciferase mRNA. The predicted RT-PCR product was 1.6 kb in size for the firefly luciferase gene in PK-15 cells infected with rPRV HN1201-EGFP-Luc, as confirmed by gel electrophoresis (Figure 2C). No specific bands of similar size were found in any of the mRNA samples of PRV HN1201 or PK-15 cells. As shown in Figure 2D, the gE protein was detected by Western blotting in extracts of PK-15 and ST cells infected with PRV HN1201, whereas no gE protein was expressed in the cells with rPRV HN1201-EGFP-Luc. However, firefly luciferase protein was detected in the PK-15 and ST cells infected with rPRV HN1201-EGFP-Luc. The results indicated the deletion of the gE gene and the presence of the firefly luciferase gene in rPRV HN1201-EGFP-Luc. In addition, gB protein expression was observed in the PK-15 and ST cells infected with both rPRV HN1201-EGFP-Luc and PRV HN1201. Firefly luciferase expression in the PK-15 cells infected with rPRV HN1201-EGFP-Luc was also corroborated by determination of firefly luciferase activity (Figure 2E).

### Biological characteristics of rPRV HN1201-EGFP-Luc

**Figure 3:**
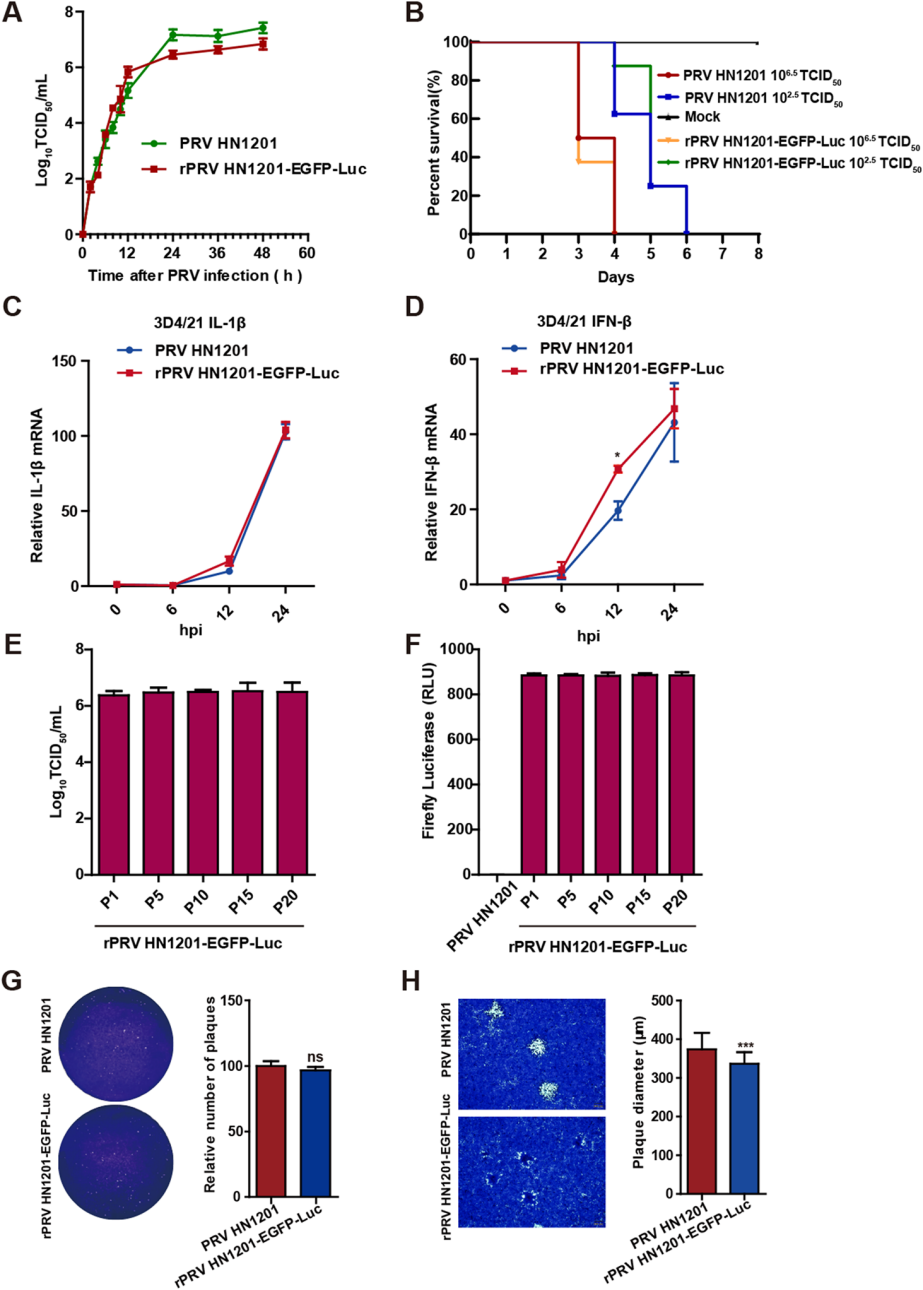
**Biological characteristics of the recombinant virus rPRV HN1201-EGFP-Luc.** **A** One-step growth curves of PRV HN1201 (MOI = 0.1) and rPRV HN1201-EGFP-Luc (MOI = 0.1) in PK-15 cells. This experiment was performed three times, and the results are shown as the mean ± SD. **B** Percentage survival of mice (eight mice per group) infected I.M. with 10^6.5^ TCID_50_ and 10^2.5^ TCID_50_ of PRV HN1201 and rPRV HN1201-EGFP-Luc. **C**, **D** Equal numbers of 3D4/21 cells were infected with PRV HN1201 or rPRV HN1201-EGFP-Luc at an MOI of 1. Total RNA was collected at 6, 12 and 24 hpi, and then, IL-1β (**C**) and IFN-β (**D**) mRNA expression was quantified by RT-qPCR. This experiment was performed three times, and the results are shown as the mean ± SD. **E** The rPRV HN1201-EGFP-Luc passage experiments were performed in PK-15 cells (passages 1–20), and then, viral titre was determined with TCID_50_ assays at specific passages (1, 5, 10, 15 and 20) of rPRV HN1201-EGFP-Luc. Data are shown as the mean ± SD from three independent experiments. **F** PK-15 cells were infected with specific passages (1, 5, 10, 15 or 20) of rPRV HN1201-EGFP-Luc (MOI = 0.1). Luciferase assays were performed in triplicate at the indicated times after infection. Data are shown as the mean ± SD from three independent experiments. **G** The plaque assay standardized on genome copy numbers. The number of rPRV HN1201-EGFP-Luc plaques was normalized to the number of PRV HN1201 plaques to detect the effect of gI/gE gene knockout on PRV packaging. Representative data from triplicate experiments are shown. Mean + SD, ns, no significant difference. **H** Plaque sizes of PK-15 cells infected with PRV HN1201 or rPRV HN1201-EGFP-Luc at 36 hpi. The mean plaque diameter of 50 plaques from one representative experiment out of three independent experiments is shown. Mean + SD, *** *P* < 0.001.

To determine whether the deletion of PRV gI/gE and the expression of EGFP and firefly luciferase influenced biological characteristics such as replication capability, lethality to mice and the ability of PRV HN1201 to stimulate cells to produce IL-1β and IFN-β, we performed one-step growth curves in virus tests, mouse survival tests and RT-qPCR. As shown in Figure 3A, the one-step growth curve of rPRV HN1201-EGFP-Luc was analogous to that of PRV HN1201 in PK-15 cells at early times, whereas the replication of rPRV HN1201-EGFP-Luc was slightly slower than that of PRV HN1201 at 24 hpi. The mouse survival test showed that the lethality of rPRV HN1201-EGFP-Luc to mice was comparable to that of PRV HN1201 (Figure 3B). RT-qPCR analysis indicated that the levels of IL-β in the 3D4/21 cells stimulated by rPRV HN1201-EGFP-Luc were similar to those in the cells stimulated by PRV HN1201 (Figure 3C). However, a significant (*P* < 0.05) increase in IFN-β expression was detected in the 3D4/21 cells stimulated by rPRV HN1201-EGFP-Luc at 12 h compared with the 3D4/21 cells stimulated by PRV HN1201. A previous study showed that PRV gE/gI is important in suppressing type I interferon in pig plasmacytoid dendritic cells (pDCs) [38]. Our study suggested that the biological characteristics of rPRV HN1201-EGFP-Luc were similar to those of PRV HN1201. However, some differences in the natural hosts (pigs) are worthy of future exploration. The viral titre and luciferase activity from rPRV HN1201-EGFP-Luc were stable through at least 20 passages (Figures 3E and F), thus indicating that rPRV HN1201-EGFP-Luc was stable.

To further investigate the effect of gI/gE gene deletion on the biological characteristics of PRV HN1201, we performed plaque assays standardized by PRV genome copy numbers to determine the number and diameter of plaques of PRV HN1201 and rPRV HN1201-EGFP-Luc. As shown in Figure 3G, the number of plaques formed by PRV HN1201 and rPRV HN1201-EGFP-Luc in PK-15 cells was not significantly different. However, the recombinant virus rPRV HN1201-EGFP-Luc formed smaller plaques than the wild-type virus PRV HN1201 (Figure 3H). These results suggested that loss of gI/gE had no significant effect on PRV particle assembly but diminished the spread of PRV between cells. Several previous studies also support this conclusion [39, 40].

### Viral titre, EGFP and luciferase activity of the recombinant virus

**Figure 4:**
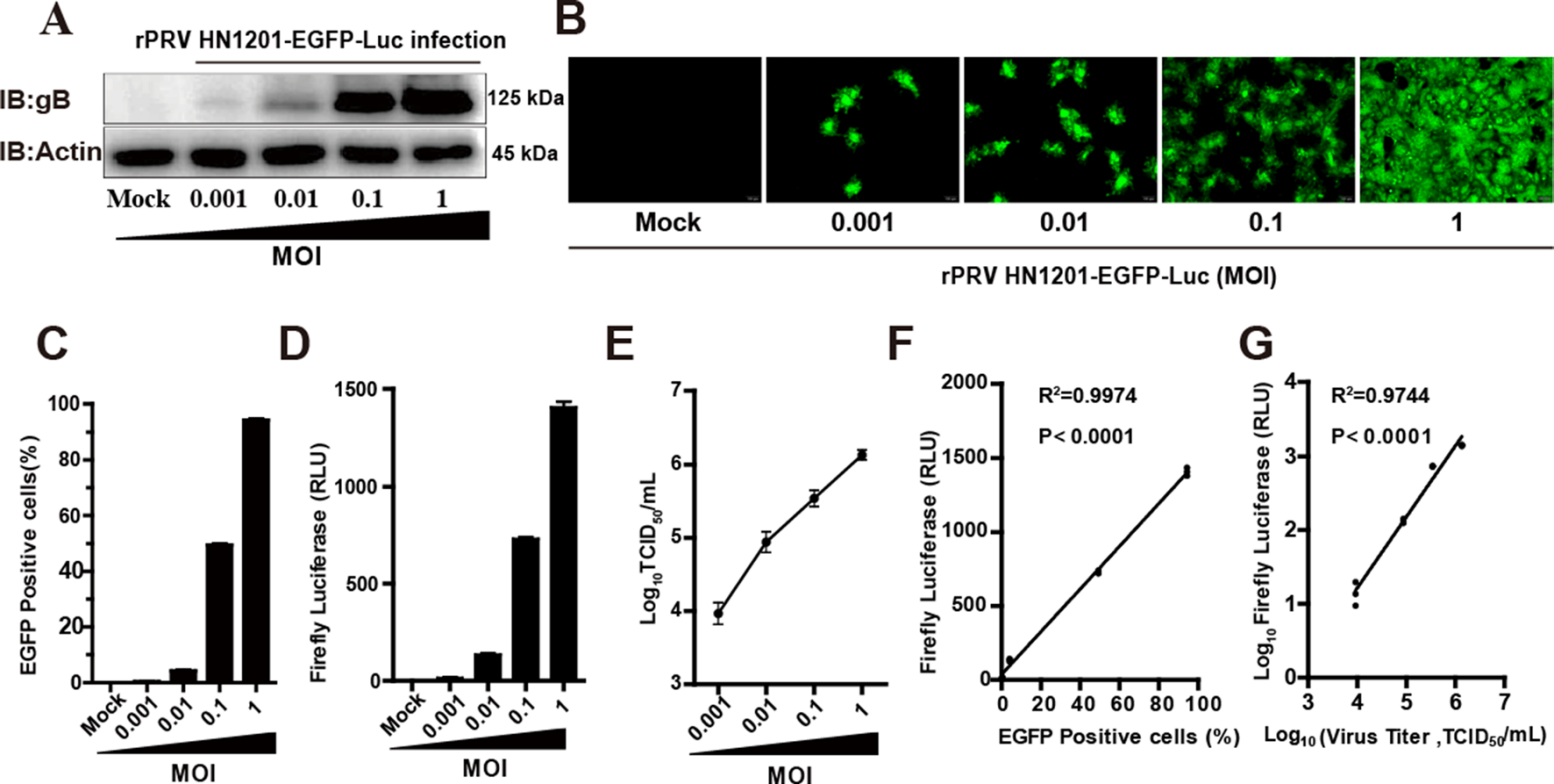
**Firefly luciferase activity, GFP expression and viral titre of recombinant virus.** **A** PK-15 cells were infected with rPRV HN1201-EGFP-Luc (MOI = 0.001, 0.01, 0.1 or 1) for 18 h and then lysed for immunoblotting analysis with antibodies against viral protein gB. β-Actin was used as the loading control. Fluorescence microscopy (**B**), fluorescence-activated cell sorting (FACS) analysis (**C**), luciferase assays (**D**) and TCID_50_ assays (**E**) of rPRV HN1201-GFP-Luc (MOI = 0.001, 0.01, 0.1 or 1) proliferation in PK-15 cells for 18 h. Data are shown as the mean ± SD from three independent experiments. Scale bar, 100 μm. **F** Correlation between luminescence intensity and GFP expression (R^2^ = 0.9974, *p* < 0.0001; GraphPad Prism 5, La Jolla, CA, USA). **G** Correlation between luminescence intensity and infectious viral titres (TCID_50_) (R^2^ = 0.9744, *p* < 0.0001; GraphPad Prism 5, La Jolla, CA, USA).

To assay the viral titre, EGFP signal and firefly luciferase activity of rPRV HN1201-EGFP-Luc could be used to evaluate the proliferation of the virus. PK-15 cells were infected with rPRV HN1201-EGFP-Luc (MOI = 0.001, 0.01, 0.1 and 1), and the EGFP signal, viral titre and luciferase activity of the virus were detected at certain time points. Western blotting and fluorescence microscopy observations showed that the signal for gB and the number of viral plaques increased with increasing viral infection in cells (Figures 4A and B). Combined with the results of flow cytometry assays (Figure 4C), luciferase activity (Figure 4D) and TCID_50_ (Figure 4E), further analysis showed that the viral titre and EGFP of the recombinant virus linearly correlated with the luciferase activity (Figures 4F and 4G). Therefore, the viral titre, EGFP signal or firefly luciferase activity of recombinant virus could be used to evaluate the replication of recombinant virus.

### In vivo tracing and distribution of recombinant virus in mice

**Figure 5:**
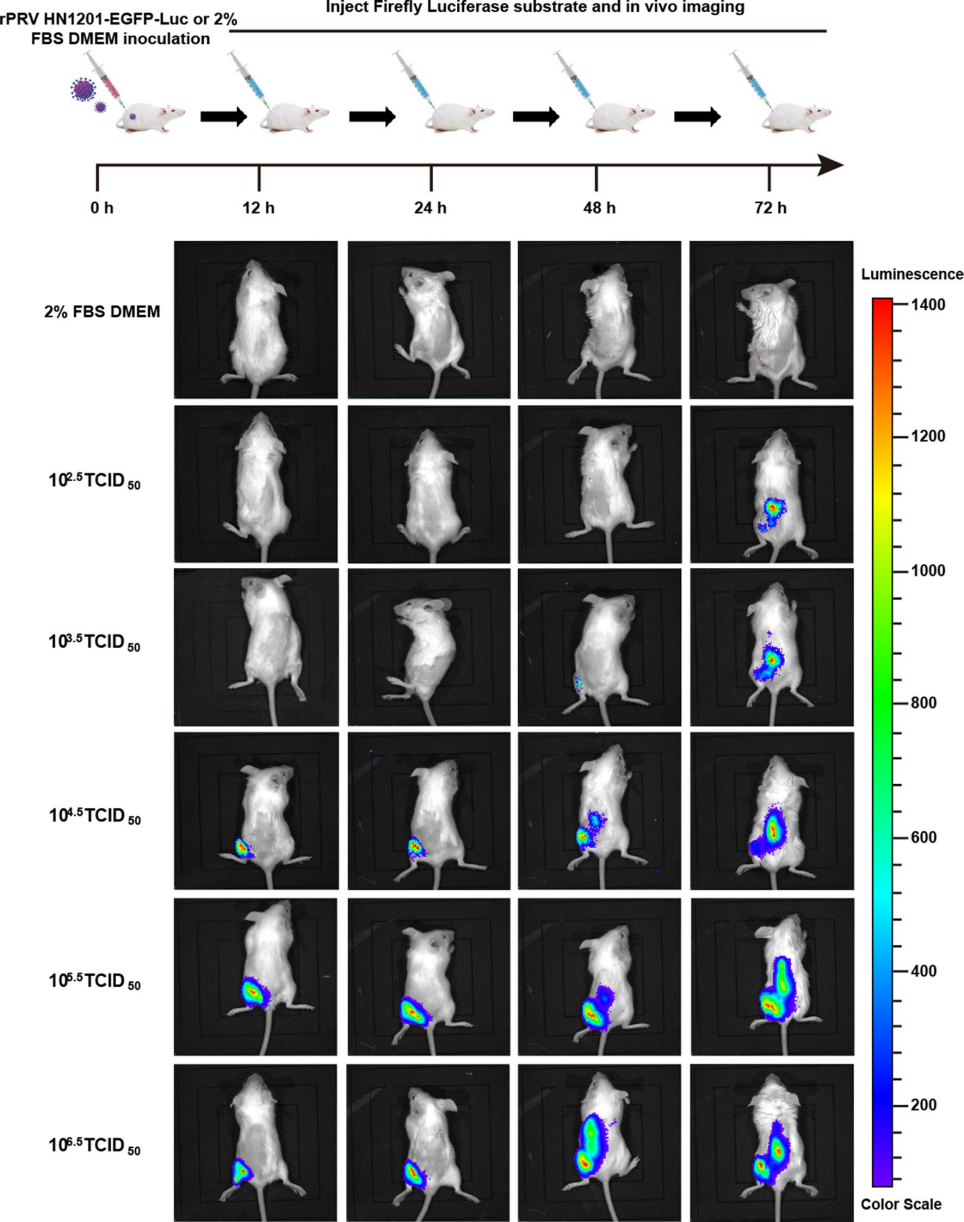
**The replication and distribution of rPRV HN1201-EGFP-Luc in a mouse model.** 6-week-old BALB/c mice were infected I.M. with 2% FBS DMEM or recombinant pseudorabies virus rPRV HN1201-EGFP-Luc (10^6.5^, 10^5.5^, 10^4.5^, 10^3.5^ or 10^2.5^ TCID_50_ per mouse) in the left leg. Viral replication and distribution were quantified at the indicated times after infection by whole-animal bioluminescence imaging.

In mice infected with 10^4.5^, 10^5.5^ or 10^6.5^ TCID_50_ recombinant virus, bioluminescence signals were detected at the injection site at 12 h and were detected at the injection site and spine at 24 h (Figure 5). Weak bioluminescence signals were detected in the mice infected with 10^3.5^ TCID_50_ recombinant virus at the injection site at 48 hpi (Figure 5). At 72 hpi, the bioluminescence signals were found mainly in the spines of the mice infected with 10^2.5^ and 10^3.5^ TCID_50_ recombinant virus, indicating that the PRV had transferred from the inoculation site to the spinal cord (Figure 5); these findings were in agreement with the mechanism of PRV infection and distribution. These results showed that the recombinant virus can be used to visualize the replication and distribution of PRV in vivo.

### The 25-HC antiviral tests in vivo and in vitro

**Figure 6:**
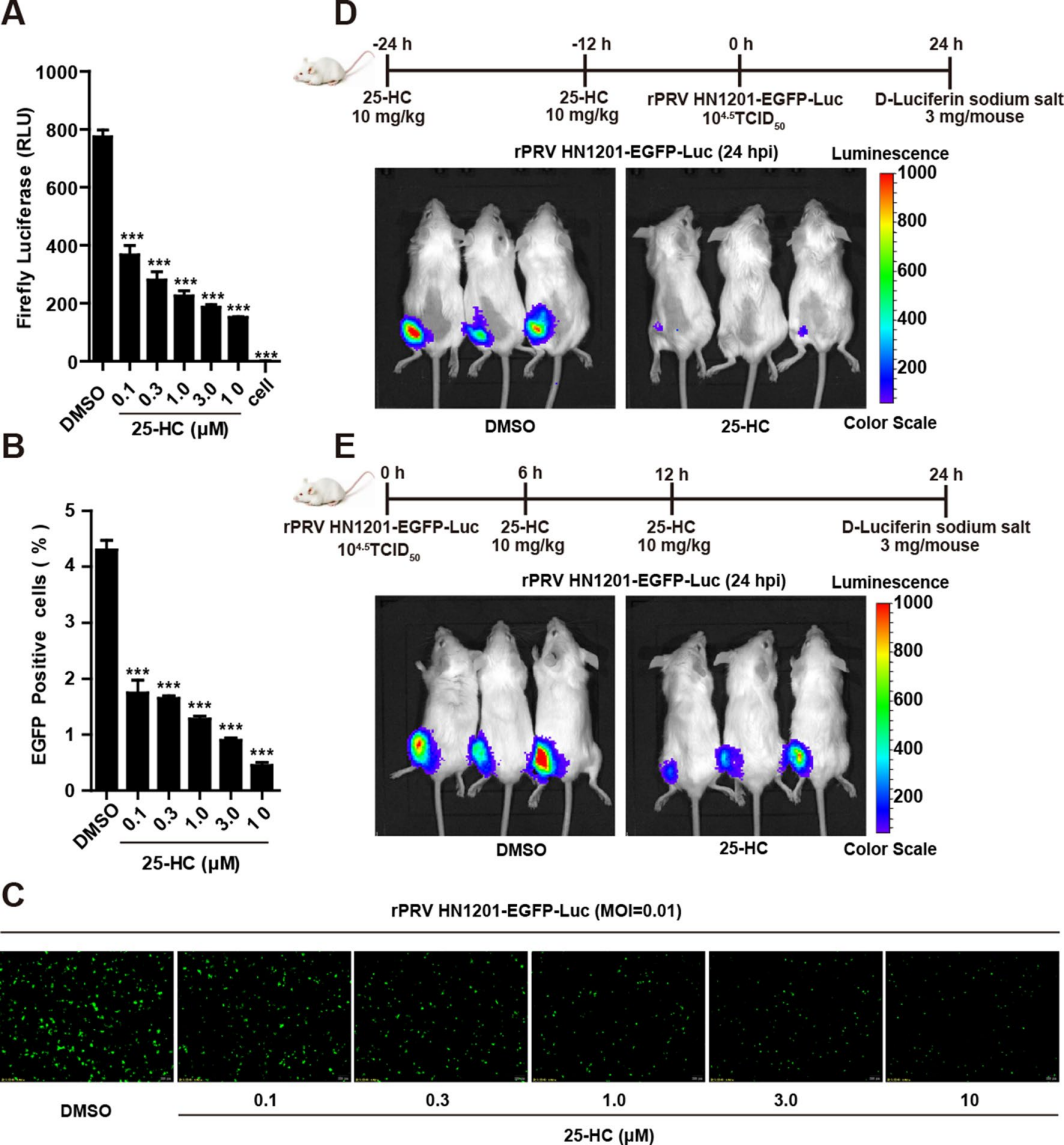
**The 25-HC antiviral tests in vivo and in vitro. Treatment with 25-HC inhibited the proliferation of rPRV HN1201-EGFP-Luc in vitro.** PK-15 cells were pretreated with the indicated concentrations of 25-HC for 4 h and then infected with rPRV HN1201-EGFP-Luc (MOI = 0.01) along with the same concentrations of 25-HC for 18 h. Luciferase assays (**A**), FACS analysis (**B**) and fluorescence microscopy (**C**) were performed. Data are shown as the mean ± SD from three independent experiments. Scale bar, 200 μm. ***P < 0.001. **D** Bioluminescent images of mice pretreated with 25-HC or DMSO at 24 h after rPRV HN1201-EGFP-Luc infection. **E** Bioluminescent images of mice in which 25-HC or DMSO was injected later upon rPRV HN1201-EGFP-Luc infection. Whole animal bioluminescence imaging was performed 24 h after rPRV HN1201-EGFP-Luc infection.

The 25-HC compound is an oxidation product of cholesterol whose formation is catalysed by cholesterol-25-hydroxylase (CH25H) [41]. This molecule not only functions as a potent oxysterol regulator of lipid metabolism [42] but also has important effects in immunity [43–46]. In a previous study, 25-HC was shown to broadly inhibit viruses, such as vesicular stomatitis virus (VSV), herpes simplex virus (HSV), human immunodeficiency virus (HIV) and murine gamma herpes virus 68 (MHV68) [47]. The inhibitory effect of 25-HC on PRV in vitro has been confirmed in our previous studies [48], but whether it has the same effect on PRV in vivo is unknown. Here, the double reporter PRV was used to confirm the effect of 25-HC on PRV reproduction in PK-15 cells and to further explore the effect of 25-HC on PRV reproduction in mice. The luciferase activity of the PK-15 cells treated with DMSO was significantly higher than that of the PK-15 cells treated with 25-HC (*P* < 0.001), and the luciferase activity of the PK-15 cells treated with 25-HC decreased in a dose-dependent manner (Figure 6A). As shown in Figure 6B, treatment with 25-HC significantly decreased the EGFP percentage in a dose-dependent manner compared with the percentages in the PK-15 cells treated with DMSO (*P* < 0.001). In addition, the fluorescence intensity in the PK-15 cells treated with 25-HC was weaker (Figure 6C). The results suggested that 25-HC inhibited the proliferation of PRV in PK-15 cells in a dose-dependent manner.

In vivo whole-animal bioluminescence imaging of the mice infected with the double reporter PRV was performed, and the 25-HC-treated group showed less bioluminescence than the DMSO group (Figure 6D), thus indicating that 25-HC also inhibited PRV in vivo. However, in a therapeutic experiment in which 25-HC was injected after PRV infection in mice, 25-HC showed a slight inhibition of PRV (Figure 6E), but this anti-PRV effect was not as pronounced as its preventive effect on PRV. Together, these results demonstrated that rPRV HN1201-EGFP-Luc is a useful tool for screening antiviral compounds both in vivo and in vitro.

## Discussion

Viral reverse genetics and the construction of reporter viruses provide a powerful tool for antiviral development and basic virology studies. Red fluorescent protein (RFP) [49, 50] and green fluorescent protein (GFP) [51] cassettes are often inserted into the PRV genome as markers, and these reporter viruses have been widely used in the screening of antiviral drugs [52] and in studies of antiviral gene function in vitro [53]. However, the viruses carrying these two markers appear to be unable to be used in antiviral studies in vivo, owing to the limited penetration ability of GFP and RFP. In this study, the recombinant pseudorabies virus rPRV HN1201-EGFP-Luc based on the variant PRV strain PRV HN1201 was developed to express both EGFP and firefly luciferase. The insertion of EGFP and the firefly luciferase expression cassette had no significant effects on viral replication (at early times), lethality to mice or the ability to stimulate 3D4/21 cells to produce IL-1β (Figures 3A–C). Although the gI and gE genes are nonessential for PRV proliferation, they play a role in the process of PRV infection of cells or animal bodies. For example, the gE/gI complex promotes cell–cell spreading and is involved in the release of the virus from cells and in cell fusion in vitro [39]. Moreover, gE-negative PRV showed a substantially diminished ability to infect second- and third-order neurons in the porcine central nervous system (CNS) [54]. The underlying molecular mechanism remains to be further explored. The PRV gene editing strategy and the recombinant virus in this study should enable convenient study of the pathogenic mechanism of PRV. The recombinant virus remained stable even after 20 passages, as confirmed by both viral titre and luciferase activity assays (Figures 3E and F). Here, we further explored the relationships among viral titre, EGFP and luciferase activity in recombinant virus-infected cells. The viral titre and EGFP of the recombinant virus were linearly correlated with the luciferase activity (Figures 4F and G), thus suggesting that the viral titre, EGFP signal or firefly luciferase activity of the recombinant virus could be used to evaluate the replication of recombinant virus.

In some previous studies, the firefly luciferase expression cassette was recombined into PRV for expression [55, 56], but few studies have reported imaging of PRV in vivo. In this study, the imaging characteristics were analysed in vivo in mice infected with different doses of the recombinant virus rPRV HN1201-EGFP-Luc. The bioluminescence signal was detected in mice as early as 12 hpi (Figure 5). Using the reporter virus, we demonstrated that 25-HC inhibited PRV replication both in vivo and in vitro. These results suggested that the reporter viruses constructed in this study may play an important role in studies of PRV mutant strains both in vivo and in vitro.

Compound 25-HC is an oxidation product of cholesterol, whose formation is catalysed by cholesterol-25-hydroxylase (CH25H). Previous studies have shown that 25-HC treatment in cultured cells broadly inhibited the growth of enveloped viruses such as herpes simplex virus (HSV) [47, 57], human immunodeficiency virus (HIV) [47, 58], Ebola virus (EBOV) [59], Nipah virus (NiV) [47] and Rift Valley fever virus (RVFV) [47]. This treatment suppresses viral growth by blocking membrane fusion between the virus and cell. Further research has shown that 25-HC directly modifies the cellular membrane, thereby inhibiting viral fusion [47]. Membrane fusion is critical for host-virus interactions, and several factors, including the viral receptors on the cell surface, the degree of curvature, the spatial conformation of membrane phospholipid bilayers, and the arrangement and mobility of phospholipid molecules, are involved in this step [60, 61]. Another study has shown that 25-HC loosens cholesterol molecules in cytomembranes, thus changing the location, arrangement and solubility of cholesterol in cytomembranes [62]. In our previous research, we demonstrated that 25-HC inhibits PRV infection by blocking the attachment and entry of the virus [48]. In addition, qPCR detection revealed that the levels of the enzymes synthesizing fatty acids (ACC, FAS and SREBP1) and cholesterol (HMGCR, HMGCS and SREBP2) decreased in 3D4/21 cells treated with 25-HC. Diminished sterol synthesis may alter the lipid distribution in the cell membrane. Cellular cholesterol metabolism is important in the viral life cycle, and the depletion of cholesterol in cell membranes prevents the formation of lipid rafts and inhibits PRV entry [63]. On the basis of the literature and our previous research, we speculate that the anti-PRV activity of 25-HC is mainly based on modulation of the biosynthesis of cholesterol or other metabolites, thus further affecting the characteristics of cytomembranes. Nevertheless, the specific molecular mechanism of 25-HC against PRV requires further study.

In previous studies, CRISPR/Cas9 technology has been widely used in viral genome editing [49, 50, 55, 64–67]. Most studies have used a single sgRNA to cleave the viral genome, and the damaged DNA is repaired via error-prone nonhomologous end joining (NHEJ) in cell repair pathways; this approach tends to produce random additions, deletions or synonymous repair at the original site [68]. A recent study used homology-directed repair following CRISPR/Cas9 cleavage to produce recombinant PRV virus [69]. In our study, a two-sgRNA CRISPR/Cas9 system was developed with two sgRNAs targeting the PRV gI and gE genes. To enable the two sgRNAs and Cas9 proteins to edit the viral genome in the same cell, we used the two-sgRNA CRISPR/Cas9 systems constructed in this study in a single plasmid system to cut the specific sites of the gI and gE genes of PRV. Then, the EGFP and firefly luciferase expression cassettes were inserted into the PRV HN1201 genome through intracellular homology-directed repair (HDR). The two sgRNA CRISPR/Cas9 systems constructed herein can be used not only to knock out large DNA fragments of PRV but also to effectively insert other foreign antigens.

The parental strain PRV HN1201 in this study is a Chinese PRV variant strain that is more pathogenic to pigs than the classic Fa strain [20]. Phylogenetic analysis illustrated that PRV HN1201 is genetically closest to the PRV human isolate hSD-1/2019 described in a recent report [25]. Hence, the reporter virus constructed on the basis of PRV HN1201 may be useful in future research.

In summary, the two-sgRNA CRISPR/Cas9 single plasmid systems used herein provided a good reference method for gene editing of PRV or any other dsDNA virus. A stable replication-competent recombinant pseudorabies virus, rPRV HN1201-EGFP-Luc carrying the EGFP and firefly luciferase expression cassettes, was generated. The reporter virus should be an excellent tool for the study of basic virology and the evaluation of antiviral compounds both in vitro and in vivo.

## Data Availability

All data and materials generated for this study are included in the article.

## Abbreviations

PRV: Pseudorabies virus
EGFP: Enhanced green fluorescent protein
PR: Pseudorabies
gE: Glycoprotein E
kb: Kilobases
HR: Homologous recombination
BAC: Bacterial artificial chromosome
CRISPR/Cas9: Clustered regulatory interspaced short palindromic repeats/CRISPR-associated protein 9
25-HC: 25-Hydroxycholesterol
DMEM: Dulbecco’s modified eagle’s medium
FBS: Foetal bovine serum
sgRNA1: Small guide RNA1
sgRNA2: Small guide RNA2
CPEs: Cytopathic effects
MOI: Multiplicity of infection
HRP: Horseradish peroxidase
PBS: Phosphate-buffered saline
PLB: Passive lysis buffer
LAR II: Luciferase assay reagent II
RLU: Relative light units
I.M.: Intramuscularly
gI: Glycoprotein I
CAG: CMV enhancer and chicken β-actin promoter
hpi: Hour post-infection
HDR: Homology-directed repair
pDC: Plasmacytoid dendritic cells
CH25H: Cholesterol-25-hydroxylase
VSV: Vesicular stomatitis virus
HSV: Herpes simplex virus
HIV: Human immunodeficiency virus
MHV68: Murine gamma herpes virus 68
RFP: Red fluorescent protein
GFP: Green fluorescent protein
NHEJ: Nonhomologous end joining
CNS: Central nervous system
EBOV: Ebola virus
NiV: Nipah virus
RVFV: Rift valley fever virus
